# Nicotinic acetylcholine receptor subunit variants are associated with blood pressure; findings in the Old Order Amish and replication in the Framingham Heart Study

**DOI:** 10.1186/1471-2350-9-67

**Published:** 2008-07-14

**Authors:** Patrick F McArdle, Sue Rutherford, Braxton D Mitchell, Coleen M Damcott, Ying Wang, Vasan Ramachandran, Sandy Ott, Yen-Pei C Chang, Daniel Levy, Nanette Steinle

**Affiliations:** 1 Department of Medicine, University of Maryland School of Medicine, Baltimore, MD, USA; 2 Department of Genetics, Southwest Foundation for Biomedical Research, San Antonio TX, USA; 3 Division of Cardiology, Boston University School of Medicine, Boston MA, USA; 4 National Heart, Lung, and Blood Institute, Bethesda, MD, USA

## Abstract

**Background:**

Systemic blood pressure, influenced by both genetic and environmental factors, is regulated via sympathetic nerve activity. We assessed the role of genetic variation in three subunits of the neuromuscular nicotinic acetylcholine receptor positioned on chromosome 2q, a region showing replicated evidence of linkage to blood pressure.

**Methods:**

We sequenced *CHRNA1*, *CHRND *and *CHRNG *in 24 Amish subjects from the Amish Family Diabetes Study (AFDS) and identified 20 variants. We then performed association analysis of non-redundant variants (n = 12) in the complete AFDS cohort of 1,189 individuals, and followed by genotyping blood pressure-associated variants (n = 5) in a replication sample of 1,759 individuals from the Framingham Heart Study (FHS).

**Results:**

The minor allele of a synonymous coding SNP, rs2099489 in *CHRNG*, was associated with higher systolic blood pressure in both the Amish (p = 0.0009) and FHS populations (p = 0.009) (minor allele frequency = 0.20 in both populations).

**Conclusion:**

*CHRNG *is currently thought to be expressed only during fetal development. These findings support the Barker hypothesis, that fetal genotype and intra-uterine environment influence susceptibility to chronic diseases later in life. Additional studies of this variant in other populations, as well as the effect of this variant on acetylcholine receptor expression and function, are needed to further elucidate its potential role in the regulation of blood pressure. This study suggests for the first time in humans, a possible role for genetic variation in the neuromuscular nicotinic acetylcholine receptor, particularly the gamma subunit, in systolic blood pressure regulation.

## Background

**Figure 1:**
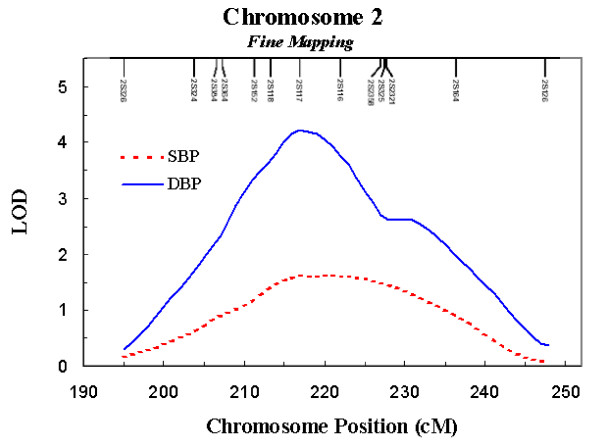
**Variance components linkage analysis of STR markers spaced approximately every 2 – 5 cM across the blood pressure linkage interval on chromosome 2q.** LOD = 4.23 (p = 0.00001) for DBP; LOD = 1.60 (p = 0.003) for SBP.

Hypertension is one of the most important risk factors for cardiovascular disease, and end-stage renal disease. Globally hypertension is ***the ***leading risk factor for morbidity and mortality [1], having multifactorial causal agents, including genetic and environmental components. We previously reported a genome wide scan of subjects from the Amish Family Diabetes study that provided strong evidence for linkage to diastolic (LOD = 3.36; p = 0.00004) and systolic (LOD = 1.64; p = 0.003) blood pressure on chromosome 2q31-q34 [2]. Follow-up analyses with additional markers resulted in an increase in the LOD score to 4.23 (p = 0.00001) for diastolic blood pressure (Figure 1). Initial fine mapping of the region with a densely spaced set of single nucleotide polymorphisms (SNPs) showed association between several SNPs in *CHRND *and systolic blood pressure (SBP). This region also contains *CHRNG *and *CHRNA1*, which encode subunits of the same nicotinic acetylcholine receptor that form the neuromuscular nicotinic acetylcholine receptor.

Systemic blood pressure is tightly regulated through sympathetic nerve activity. Feedback information about systemic blood pressure is conveyed to the brainstem by baroreceptor afferents. Accumulating evidence suggests that perturbations in these neuronal mechanisms may play a role in hypertension [3]. Indeed, hypertensive individuals tend to have increased sympathetic nerve activity [4,5] and a functional change in the sensitivity of the baroreceptor reflex [6]. In the spontaneously hypertensive rat, abnormalities in cholinergic activity were shown to be involved in the development of hypertension [7]. Nicotinic acetylcholine receptors play an important role in processes involving regulation of systemic blood pressure [7], and may also play an important role in neural developmental processes including cell differentiation, outgrowth and survival [8].

The nicotinic acetylcholine receptor has two main receptor subtypes, the neuronal (ganglionic) receptor found in the central nervous system and the neuromuscular receptor found in the neuromuscular junction of somatic muscle. The neuromuscular nicotinic acetylcholine receptor consists of two alpha subunits, a beta, a delta and either a gamma subunit in fetal muscle or an epsilon subunit in adults [9-11] Polymorphisms in the alpha and delta subunits have been shown to confer increased risk of myasthenia gravis [12-15].

The gamma subunit of the nicotinic acetylcholine receptor is reported to be important for neuromuscular development [16] and ligand binding [17]. Disruption of the gamma subunit, *in vitro*, prevents the correct localization of the receptor in cell membranes [18]. The gamma subunit contributes to neuromuscular signal transduction and is also important for neuromuscular organogenesis [16,19]. Rare mutations in *CHRNG *have been shown to cause Escobar Syndrome, a condition manifested by orthopedic and cranial anomalies [18,20].

To identify common genetic variants in the 3 nicotinic acetylcholine receptor genes on chromosome 2q, each gene was sequenced in 24 subjects enrolled in the Amish Family Diabetes Study (AFDS). Variants were then genotyped in the entire AFDS sample and tested for association with blood pressure levels. Associated variants were genotyped in an outbred Caucasian population from the Framingham Heart Study as a replication sample. To assess whether associations relate to regulation by the postganglionic nicotinic acetylcholine receptor, we genotyped replicated variants in a second sample of Amish and tested for association with response to a cold pressor stress test. We provide the first evidence that a common genetic variant in *CHRNG *is associated with systolic blood pressure.

## Methods

### Samples

#### Primary Amish Sample

Our principle sample consisted of Old Order Amish (OOA) individuals from Lancaster County, PA. The OOA are a genetically closed founder population originating from Western Europe and who immigrated to central Pennsylvania in the early 1700s [21]. They are particularly well suited for genetic studies of complex diseases and traits because they maintain a relatively uniform lifestyle and tend not to use prescription medications. Family members tend to eat meals together and the Amish diet is largely similar across families. In addition, farming is still the primary occupation and most OOA maintain a lifestyle of high physical activity levels. The homogenous lifestyle of the Amish reduces the variability of many environmental influences and thereby enhances the power to detect the genetic susceptibility to complex diseases.

Our primary Amish sample consisted of 1,189 participants of the AFDS. The AFDS began in 1995 with the goal of identifying susceptibility genes for type 2 diabetes and related traits [22]. Probands were individuals identified with type 2 diabetes and family members 18 years of age and over were recruited into the study. The 1,189 individuals recruited into the study were members of 226 pedigrees. The study protocol was performed either in the individual's home or in the Amish Research Clinic in Strasburg, Pennsylvania. Multiple phenotypes, including blood pressure measurements, were obtained using a standardized protocol. After the subject rested for at least 5 minutes, a single observer recorded systolic (first phase) and diastolic (fifth phase) blood pressure (SBP and DBP respectively) to the nearest mmHg in duplicate using a standard sphygmomanometer.

The use of anti-hypertensive medication was <10% among the AFDS subjects. We accounted for hypertension medication use in two ways. First we performed the analysis using only those subjects who were not prescribed anti-hypertension medication. This approach has the possibility of biasing the results because we are removing those with the highest (untreated) blood pressures from the analysis. Since the use of anti-hypertension medication is not as common in the Amish as the general population, this bias is reduced. Second, we adjusted for medication use by adding a constant to the observed blood pressure measurements, 10 mmHg to SBP and 5 mmHg to DBP. This approach was suggested by Cui et. al. [23] and later validated by Tobin et. al. [24].

#### Framingham Heart Study Sample

We genotyped BP-associated variants in a replication sample from an independent Caucasian population from Framingham, MA. The Framingham Heart Study began in 1948 to investigate the causes of heart disease [25]. Men and women (n = 5,209) between the ages of 28 and 62 years were recruited and followed prospectively over time. Beginning in 1971, offspring of the original cohort were recruited as part of the Framingham Offspring Study [26]. An additional 5,124 individuals were recruited for this study, consisting of offspring and spouses of offspring of the original cohort. Our sample consisted of 1,839 unrelated individuals who were randomly selected from the Framingham Offspring Study as per the protocol established by the collaboration between the National Heart, Lung, and Blood Institute and Boston University. Each participant underwent a physician administered exam where blood pressure measurements were taken and a DNA sample was collected. Data on blood pressure measurements were collected every four years for members of the offspring cohort. For the analysis in this report, we used data from visits which took place from February 1995 to September 1998. The participants ranged in age from 29 to 86 years at the time of this visit. Of the 1,839 randomly selected individuals, 1,759 have both a SBP and DBP measurement recorded. Anti-hypertension medication use in the Framingham cohort was more common (29.6% in our sample) and was accounted for by the methods described above.

#### Amish Sample – Cold Pressor Test

Variants associated with blood pressure in both the initial Amish sample and the Framingham sample were genotyped in a second sample of Amish who were participants of the Heredity and Phenotype Intervention (HAPI) Heart Study. The HAPI Heart Study was initiated to identify genes that interact with specific environmental exposures to influence cardiovascular risk factors [27]. As part of this study, we administered a 2.5-minute cold pressor test (CPT) to 851 participants from 18 extended, Old Order Amish families from Lancaster County, Pennsylvania. Of the 851 participants of the CPT in the HAPI Heart Study, 149 were also participants of the AFDS. In the HAPI Heart study, we utilized repeated blood pressure measurements taken before, during, and after the CPT to derive physiologically relevant response traits such as reactivity to and recovery from the cold pressor stimulus. Individuals remained semi-reclined until their blood pressure was stable. The right hand and wrist to the ulnar styloid was submerged in ice water for 2.5 minutes. Blood pressure and heart rate were measured at 0, 1, 2, 3, 4, 5, 7.5, 10, 15, and 20 minutes by use of an appropriate fitting automated blood pressure cuff. CPT reactivity was defined as the change in blood pressure levels from baseline to 2 minutes after immersion. CPT recovery was defined as the change in blood pressure from 2 minutes to 5 minutes after immersion. The change in blood pressure (slope) was calculated as sum((BP-mean(BP))(t-mean(t))/sum((BP-mean(BP))^2^) for all non-missing blood pressure measurements. All subjects were withdrawn from their medications (only 2 subjects were on anti-hypertensive medications) for 7 days prior to testing.

The Amish Studies and FHS were approved by respective Institutional Review Boards, and conform to the Declaration of Helsinki. All subjects provided informed consent.

#### DNA Sequencing and Genotyping

Genetic variants in *CHRNA1*, *CHRND *and *CHRNG *were identified through direct sequencing of 24 non-first degree relatives from the AFDS. This sequencing set provides 90% power to detect at least one copy of the minor allele for single nucleotide polymorphisms (SNPs) with allele frequencies of greater than or equal to 5%. We sequenced all exons, splice junctions (~100 bp of the intronic flanking sequence), and untranslated regions. Both strands were sequenced on an ABI 3730 DNA sequencer and analyzed using Sequencher V4.5 (Gene Codes Corporation, Ann Arbor, MI). Initial sequencing identified 20 variants, 4 of which were not annotated with rs numbers in dbSNP and were subsequently not confirmed. (Sequence and primer information is available at . Follow the links "Publication List" and "Supplemental Data.") Three SNPs (rs10629807, rs260068, rs11674608) were in perfect linkage disequilibrium in the sequencing set of 24 individuals with three other SNPs identified (rs2646165, rs2646159, rs2697782 respectively) and therefore represented redundant information. Only the later set of SNPs were genotyped in the complete sample. One SNP (rs2697782) failed our quality control procedures. Thus genotypes for 12 SNPs were available for association testing in the complete AFDS sample. Genotyping was carried out using the manufacturer's protocol on the SNPstream Ultra High Throughput platform (Beckman Coulter, Fullerton, CA) and the Pyrosequencing PSQ HS 96A system (Pyrosquencing, Uppsala, Sweden). The genotype error rates based on blind replicates were 0 – 2%. All genotype frequencies conformed to Hardy-Weinberg expectations with the exception of rs3762528 (p = 0.02).

### Statistical Methods

Analysis performed on the genotypes for the AFDS and HAPI Heart Study utilized a variance component framework, which modeled the genotype as a fixed effect and controlled for sex, age and age squared. Each variant's additive, dominant and recessive effects were estimated using separate regression models. The variance component approach models the correlations between the trait of interest (the dependent variable) and dummy variables coding the genotype effect (independent variable) conditional on fixed covariates and the residual correlations among individuals implied by the pedigree structure. Specifically, the covariance between each pair of individuals within the pedigree was estimated as a function of their degree of relationship, the trait heritability, and the phenotypic variance of the trait. The analysis was performed using the SOLAR software package [28].

Simple linear regression was used to test for the association between genotype and blood pressure in the Framingham sample. Three genetic models (additive, dominant and recessive) were tested regardless of which model was identified in the OOA.

An alpha level of < 0.01 was considered as strong evidence of association.

## Results

### Sample Characteristics

Characteristics of the three samples are shown in Table 1. The average age of the primary sample, the AFDS, was 49.4 years, while the mean age of the Framingham participants was 59.4 years. The use of anti-hypertensive medication in the OOA sample was rare (9.3%) as compared to the population based sample from FHS (29.6%). The AFDS sample contained 139 (11.7%) individuals with diabetes. The participants in the HAPI Heart Study tended to be younger (mean age = 43.6 years) and were relatively more healthy (lower BMI and resting blood pressure) than either the AFDS or FHS.

**Table 1:** Sample characteristics for the AFDS, FHS, and the HAPI Heart Study.

	AFDS	FHS	HAPI
N	1189	1759	851
Sex (% male)	45.3%	49.6%	53.2%
Mean (SD) age (yrs)	49.4 ± 17.1	59.4 ± 9.4	43.6 ± 13.9
Mean (SD) BMI (kg/m^2^)	27.2 ± 5.0	28.0 ± 5.1	26.7 ± 4.5
Hypertension medication use (%)	9.3%	29.6%	*
Mean (SD) SBP/DBP (mmHg)	122.2 ± 15.9	125.5 ± 17.4	116.5 ± 13.3
Those on medication removed	77.7 ± 9.6	74.8 ± 9.1	70.6 ± 7.5
Mean (SD) SBP/DBP (mmHg)	125.2 ± 18.9	132.4 ± 20.8	*
Constant added to those on medication	78.5 ± 10.2	77.0 ± 10.0	

* Anti-hypertension medications were withdrawn 7 days prior to testing and therefore no HAPI subjects were on medications at the time of study.

### Linkage Disequilibrium Block Structure

**Figure 2:**
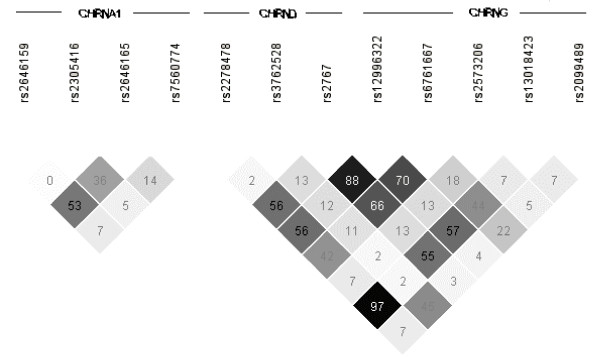
**Linkage disequilibrium block structure for *CHRNA1*, *CHRND *and *CHRNG *in the Old Order Amish.** Values given are r-squared times 100.

Figure 2 shows the linkage disequilibrium (LD) plots for variants genotyped in the complete AFDS sample. There is little LD between the four SNPs in *CHRNA1*. *CHRNG *SNP rs12996322 is correlated with rs6761667 (r^2 ^= 0.70) and in strong LD with *CHRND *SNP rs2767 (r^2 ^= 0.88). SNPs rs12996322 and rs2767 span the inter-genetic region between *CHRND *and *CHRNG*. There is also significant long range correlation between *CHRND *SNP rs2278478 and *CHRNG *SNP rs13018423 (r^2 ^= 0.97), which are separated by 16.8 kb.

### Association with Blood Pressure

Table 2 shows the results from the association analysis to blood pressure adjusted for age, sex and familial relatedness in the AFDS sample. The values given are p-values for the association analysis after removing those on anti-hypertensive medication from the analysis. Very similar results were found when accounting for medication in the analysis as described in the methods (data not shown). Evidence of association with blood pressure was present for several variants including 6 variants whose most significant model had strong evidence for association (p ≤ 0.01) with either SBP or DBP (Table 2). As shown in Figure 2, these associations may not be independent signals as some of the associated SNPs are in linkage disequilibrium.

**Table 2:** Association analysis of polymorphisms in *CHRNA1*, *CHRND *and *CHRNG *with blood pressure in the AFDS.

Marker	Polymorphism	Minor Allele Frequency	Mean SBP/DBP By Genotype (mmHg)			P-Value	
			Homozygous for major allele	Heterozygous	Homozygous for minor allele	SBP	DBP

*CHRNA1*							
rs2646159	Intron 2	0.09	122/77	122/79	132/91	0.14	0.009
rs2305416	Exon 7: His320His	0.06	123/78	123/77	134/79	0.09	0.70
rs2646165	Intron 4	0.14	123/76	123/79	124/81	0.24	0.07
rs7560774	Intron 1	0.46	122/79	122/77	123/77	0.23	0.04
							
*CHRND*							
rs2278478	Intron 2	0.29	122/77	123/78	123/80	0.32	0.45
rs3762528	Intron 9	0.09	121/77	126/79	113/78	0.0007	0.18
rs2767	Exon 12 UTR	0.43	121/79	125/80	128/81	0.0007	0.05
							
*CHRNG*							
rs12996322	Intron 1	0.41	120/77	124/78	126/79	0.007	0.26
rs6761667	Intron 6	0.48	119/76	123/78	125/78	0.0001	0.007
rs2573206	Intron 8	0.19	120/77	121/77	123/78	0.03	0.04
rs13018423	Intron 8	0.29	122/77	123/78	125/78	0.31	0.63
rs2099489	Exon 12: Arg474Arg	0.20	121/77	124/78	127/78	0.0009	0.02

Values given are p-values from the most significant genetic model (additive, dominant, recessive), controlling for age and sex. Results are given after removing those on antihypertensive medication from the analysis.

Five BP-associated variants (rs2646159, rs3762528, rs2767, rs12996322, and rs2099489) were genotyped in the Framingham sample and association results are shown in Table 3. The association with rs2099489 was the only SNP showing significant association with blood pressure in the Framingham sample. This SNP is a C -> T (Arg_474 _-> Arg_474_) synonymous coding polymorphism in the last exon of *CHRNG*.

**Table 3:** Replication of association in a sample of Caucasians enrolled in the FHS.

Marker	Minor Allele Frequency	Mean SBP/DBP By Genotype (mmHg)			P-Value	
		Homozygous for major allele	Heterozygous	Homozygous for minor allele	SBP	DBP

rs2646159	0.07	126/75	124/75	127/68	0.12	0.14
rs3762528	0.06	125/75	127/75	137/81	0.30	0.25
rs2767	0.36	126/75	125/75	125/74	0.24	0.72
rs12996322	0.36	125/75	126/75	126/74	0.11	0.53
rs2099489	0.20	125/75	125/75	133/75	0.009	0.25

* Values given are p-values from the most significant genetic model (additive, dominant, recessive), controlling for age and sex. Results are given after removing those on antihypertensive medication from the analysis.

Although not statistically significant, the rs2099489 SNP was modestly associated with baseline SBP in the HAPI Heart Study (p = 0.29). However, the allele associated with higher mean SBP levels in AFDS and FHS was also associated with higher mean SBP levels in HAPI. After taking into account individuals that were in both the AFDS and HAPI cohorts, we combined the Amish samples and found rs2099489 remained strongly associated with SBP (p = 0.008). There was no association between rs2099489 and any of the response traits (change in blood pressure during the reactivity time period, change in blood pressure during recovery time period) to the cold pressor test.

**Figure 3:**
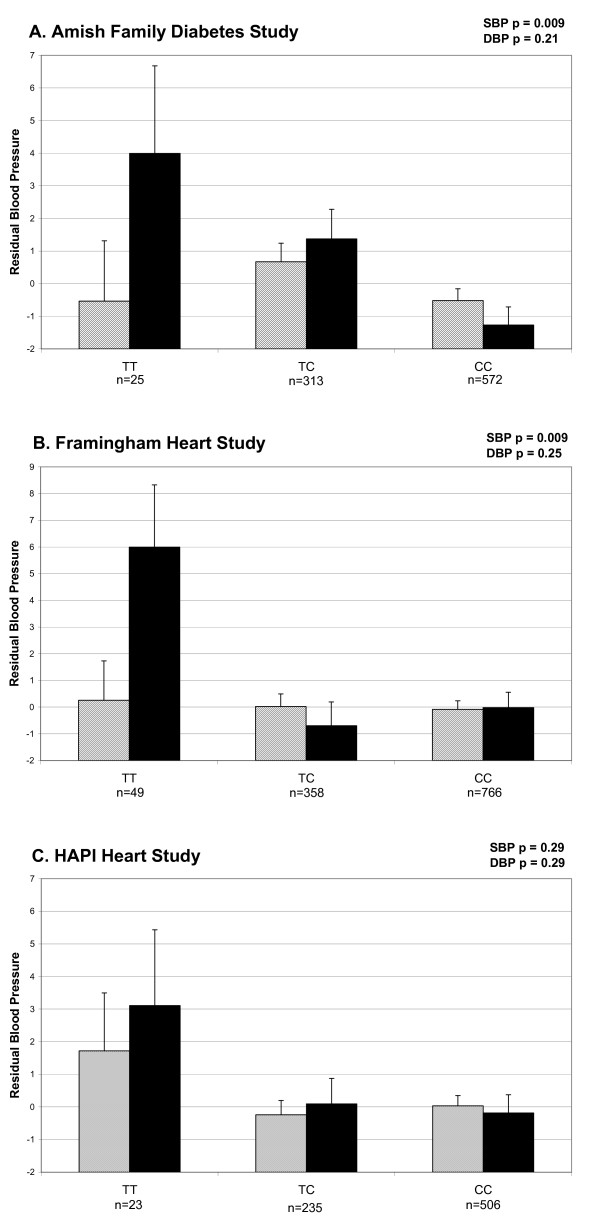
**Mean of age and sex adjusted residual blood pressure by rs2099489 genotype for the A. Amish Family Diabetes Study; B. the Framingham Heart Study; and C. the Heredity And Phenotype Intervention (HAPI) Heart Study.** SBP residual in solid bars, DBP residual in hatched bars. Standard error is represented by vertical lines.

Figure 3 shows the distribution of blood pressure by rs2099489 genotypes in the AFDS, FHS and the HAPI Heart Study samples. In both the OOA and FHS populations, the individuals with the TT genotype at SNP rs2099489 have the highest age and sex adjusted residual SBP. The TT genotype is associated with a 3 – 6 mmHg increase in blood pressure over the age and sex predicted values. This effect of 3 – 6 mmHg remained in each sample after adjusting for BMI which provides evidence that rs2099489 is acting in pathways not mediated by body mass.

## Discussion

Evidence from animal models supports the role of the nicotinic acetylcholine receptor in blood pressure regulation in the central nervous system. The potential role in blood pressure regulation of acetylcholine receptors at the neural muscular junction remains to be elucidated. It has been shown that spontaneously hypertensive rats, have a lower number of nicotinic acetylcholine receptors in spinal cord membranes than age-matched normotensive rats [29] Furthermore, studies have shown rats with varying blood pressure levels maintain relatively constant numbers of nicotinic receptors. This indicates that blood pressure does not drive receptor number but rather that the lower receptor number might be a primary event in hypertension [30].

We investigated the association between blood pressure regulation and genetic polymorphisms in genes encoding three subunits of the neuromuscular nicotinic acetylcholine receptor located on chromosome 2q. We demonstrated significant association with DBP and genetic variation in *CHRNA1 *and between SBP and several polymorphisms in *CHRND *and *CRHNG *in OOA subjects from the AFDS. However, blood pressure-associated variants in *CHRNA1 *and *CHRND *were not replicated in a sample from the Framingham Heart Study, perhaps indicating that these associations were false positive signals or, less likely, that these variants are only relevant to blood pressure determinants in the Amish. By contrast, we did demonstrate replication of the association of the *CHRNG *SNP rs2099489 with SBP. The replicated rs2099489 is a C-> T synonymous coding SNP in the last exon of the gamma subunit of the nicotinic acetylcholine receptor. In our Caucasian populations, the TT genotype at this locus was associated with 3–6 mmHg higher SBP than age and sex predicted values. A trend with a similar effect size for association between SBP and rs2099489 was detected in an independent OOA cohort from the HAPI Heart Study. Although not of statistical significance in the HAPI cohort, the fact that the subjects are on average younger and healthier than subjects in the AFDS may influence the association result for rs2099489 in this cohort. To place our findings in the context of potential impact on general health, we estimated the change in 10 year risk of myocardial infarction or death using the Framingham Risk score calculator available online through the National Heart, Lung, and Blood Institute . For a man age 55 years or older, or a woman age 65 or older who has normal cholesterol and does not use tobacco and is not currently taking medication to lower blood pressure, each incremental rise in SBP of 5 mm Hg would increase the 10 year Framingham Risk of myocardial infarction or death by approximately 1%.

Although the polymorphism we identified is synonymous, it may influence expression by altering pre mRNA splicing, mRNA stability, or efficiency of translation [31]. Alternatively, rs2099489 may not be causative but rather may be in linkage disequilibrium with a causative, as yet, ungenotyped polymorphism. While this is possible, it is unlikely that such a polymorphism exists in a coding region since all exons were sequenced. Further, it is possible that the association identified is a false positive. One reason for false positives is population stratification and is unlikely to be a significant concern in this study because we are using relatively homogenous Caucasian populations, particularly the Amish, whose ancestry since emigration to the New World is well characterized. The replication of our findings for rs2099489 in two distinct populations is further evidence against a false positive finding.

Hales and Barker posed the notion that fetal genotype and intra-uterine environment influence susceptibility to chronic diseases later in life [32]. Our findings are consistent with this hypothesis, demonstrating association between genetic variation in a gene expressed during embryogenesis and adult hypertension. Additional work will be necessary to discern how this receptor subunit, currently understood to be expressed during fetal development, impacts blood pressure in adulthood. It is also possible that this receptor may be expressed during adulthood at levels that are below current detection thresholds, or expressed transiently during periods of metabolic stress. Neoteric models to test these hypotheses will need to be developed to further our understanding of the role of *CHRNG *in blood pressure regulation.

## Conclusion

Our study suggests for the first time in humans, a possible role for genetic variation in the neuromuscular nicotinic acetylcholine receptor, particularly the gamma subunit, in SBP regulation. Additional analyses of these polymorphisms in other ethnic populations, as well as studies of the effect of genetic polymorphisms on expression and function of this nicotinic acetylcholine receptor are needed to further elucidate its potential role in the regulation of blood pressure.

## Competing interests

The authors declare that they have no competing interests.

## Authors' contributions

PFM, SR, YW, CMD and SO carried out the molecular genetic studies, and participated in the sequencing. PFM drafted the manuscript. Y–PCC and BDM participated in the design of the study. PFM and BDM performed the statistical analysis. NS conceived of the study, and participated in its design and coordination and provided significant contributions toward writing the manuscript. DL and VR led the studies in the Framingham cohort and provided guidance in the approach to the analysis in the Framingham cohort. All authors read and approved the final manuscript.

## Pre-publication history

The pre-publication history for this paper can be accessed here:


